# Associations of Dietary Intakes of Total and Specific Types of Fat with Blood Lipid Levels in the Filipino Women’s Diet and Health Study (FiLWHEL)

**DOI:** 10.5334/gh.1209

**Published:** 2023-06-12

**Authors:** Heejin Lee, Hyojin Kim, Sherlyn Mae P. Provido, Minji Kang, Grace H. Chung, Jae W. Lee, Sangmo Hong, Sung Hoon Yu, Chang Beom Lee, Jung Eun Lee

**Affiliations:** 1 Department of Food and Nutrition, College of Human Ecology, Seoul National University, Seoul, Republic of Korea; 2 Research Institute of Human Ecology, Seoul National University, Seoul, Republic of Korea; 3 Department of Food and Nutrition, Duksung Women’s University, Seoul, Republic of Korea; 4 Child Development and Family Studies, College of Human Ecology, Seoul National University, Seoul, Republic of Korea; 5 Department of Computer Science and Engineering, Seoul National University, Seoul, Republic of Korea; 6 Division of Endocrinology and Metabolism, Department of Internal Medicine, Hanyang University Guri Hospital, Hanyang University College of Medicine, Guri, Republic of Korea

**Keywords:** Dyslipidemia, fat intake, Filipino women

## Abstract

**Background:**

Limited evidence exists on the association between dietary fat intake and lipid profiles in Southeast Asian populations.

**Objectives:**

We aimed to examine the cross-sectional associations of dietary intake of total and specific types of fat with dyslipidemia in Filipino immigrant women in Korea.

**Methods:**

We included 406 Filipino women married to Korean in the Filipino Women’s Diet and Health Study (FiLWHEL). Dietary fat intake was assessed using 24-hour recalls. Impaired blood lipid profiles were defined as high total cholesterol (TC) (≥200 mg/dL), high triglyceride (TG) (≥150 mg/dL), high LDL Cholesterol (LDL-C) (≥ 130 mg/dL), or low HDL cholesterol (HDL-C) (<50 mg/dL). The genomic DNA samples were genotyped using DNA chip. The odds ratios (ORs) and 95% confidence intervals (CIs) were calculated using multivariate logistic regression.

**Results:**

Substituting carbohydrates with dietary saturated fat (SFA) intake was associated with increased prevalence of dyslipidemia; ORs (95% CIs) for subsequent tertiles compared to the first tertile were 2.28 (1.19–4.35), and 2.88 (1.29–6.39) (*P for trend* = 0.02). When we examined individual markers, ORs (95% CIs, *P for trend*) comparing the third to the first tertile were 3.62 (1.53–8.55, 0.01) for high TC, 1.46 (0.42–5.10, 0.72) for high TG, 4.00 (1.48–10.79, 0.02) for high LDL-C, and 0.69 (0.30–1.59, 0.36) for low HDL-C. When we examined the interaction by LDL-C-related polymorphisms, the association with dyslipidemia was more pronounced among participants with CC alleles than among those with T alleles of rs6102059 (*P for interaction* = 0.01).

**Conclusions:**

High dietary SFA intake was significantly associated with a high prevalence of dyslipidemia in Filipino women in Korea. Further prospective cohort studies are warranted to determine risk factors for CVD in Southeast Asian populations.

## Introduction

Cardiovascular disease (CVD) is the primary cause of death worldwide [1] and in the Philippines [2]. The Global Burden of Disease Study estimated that 10 million deaths and 207 million disability-adjusted life-years (DALYs) of CVD were attributable to dietary risk factors in 2017, indicating that CVD was the highest cause of diet-related deaths [3].

Total fat and specific types of fats have long been investigated in animal and human studies. The differences between saturated and unsaturated fats, including structure, oxidation reactions, and major food sources, have been suggested to confer different effects on vascular health. Replacing saturated fat (SFA) intake with unsaturated fat intake has been recommended for CVD prevention [4]. Evidence concerning the benefit of monounsaturated fat (MUFA) intake for cardiovascular health has been inconsistent [5], whereas polyunsaturated fat (PUFA) intake showed an association with CVD risk reduction compared with MUFA intake [6]. However, most of the evidence was based on Western studies, and limited studies were conducted in Southeast Asian populations.

Carbohydrate intake is greater in Asia than in Western countries, accounting for approximately 55–65% of the total energy intake in Asia [7,8]. However, dietary fat intake gradually increases in Asia [9,10,11], and Southeast Asians consume high SFAs relative to other Asian populations [12]. Therefore, we examined the associations between dietary intake of total and specific types of fats and dyslipidemia, an intermediate marker of CVD risk, in Filipino immigrant women in Korea.

## Methods

### Study population

The Filipino Women’s Diet and Health Study (FiLWHEL) participants were recruited from March 2014 to April 2016. Details on the FiLWHEL study have been described elsewhere [13]. In brief, 504 women who had been married to Koreans and aged older than 19 years were initially recruited. Information on demographics, socioeconomic status, medical history, anthropometric features, and health-related behaviors was collected through self- or interviewer-administered questionnaires. Fasting blood samples were collected on-site.

This study was approved by the Institutional Review Board (IRB) of Sookmyung Women’s University (SMWU-1311-BR-012), and performed following the 1964 Helsinki declaration. All participants provided written informed consent.

### Dietary assessment

Dietary information was obtained using the one-day 24-hour recall method. Dietary intakes of foods and nutrients were calculated using computer aided analysis program (CAN-pro 4.0, Korean Society of Nutrition, Seoul, Korea). Energy-adjusted nutrient intake was obtained following the residual method [14]. The percent energy of nutrients was calculated by dividing fat intake converted to calories by the total energy intake and multiplying the quotient by 100. Each energy-adjusted or percent energy from fat intake (total fat, SFA, MUFA, and PUFA) was categorized into tertiles. We calculated the top five contributing foods of individual nutrients based on the ingredients from the recipes reported.

### Ascertainment of outcomes

Participants fasted for at least eight hours before blood collection. Blood samples were collected by a professional phlebotomist under physician supervision. The blood samples were immediately centrifuged, aliquoted, and analyzed for a few biomarkers. The primary outcomes of this study were circulating levels of serum fasting lipid profiles: total cholesterol (TC), triglyceride (TG), low-density level cholesterol (LDL-C), and high-density level cholesterol (HDL-C). Total cholesterol, TG, and HDL-C were analyzed using the enzymatic calorimetric method (Seegene Medical Foundation Laboratory, Seoul, Korea) with a Cobas 8000 C702-1 analyzer (Roche Diagnostics, Basel, Switzerland). The inter-assay coefficients of variation for TC, TG, and HDL-C ranged from 1.40–2.99%, 1.48–2.45%, and 0.98–2.43%, respectively. We calculated LDL-C levels using the Friedewald equation ‘LDL-C = TC – HDL-C – TG/5 (all units are in mg/dL)’ [15]. The outcomes were dichotomized as low and high lipid levels: TC (0 <TC < 200, TC ≥ 200 mg/dL), TG (0 < TG < 150, TG ≥ 150 mg/dL), and LDL-C (0 < LDL-C < 130, LDL-C ≥ 130 mg/dL) based on the borderline values from the National Cholesterol Education Program (NCEP) Adult Treatment Panel III (ATP III) [16]. Because of the small number of participants with HDL-C levels < 40 mg/dL (n = 28), we adopted the cutoff value of metabolic syndrome for low HDL-C (0 < HDL-C < 50, HDL-C ≥ 50 mg/dL) [16]. Herein, we defined dyslipidemia as TC ≥ 200 mg/dL or one or more of TG ≥ 150 mg/dL or LDL-C ≥ 130 mg/dL. Homeostatic Model Assessment of Insulin Resistance (HOMA-IR) was calculated by using the equation ‘fasting serum insulin (μU/ml) × fasting plasma glucose (mmol l^–1^)/22.5’ [17].

### Genotyping

Collected genomic DNA (gDNA) samples were genotyped using KoreanChip, Axiom™ KORV1.1 96-Array Plate (TheragenBio Co., Ltd., Seongnam, Korea) [18]. We genotyped 374 gDNA samples available in this study. Regarding quality control, we excluded SNPs with a low genotype call rate (<0.05), deviating from Hardy-Weinberg equilibrium (HWE < 1 × 10^–6^), and low minor allele frequency (MAF < 0.01). A meta-analysis previously reported 11 single nucleotide polymorphisms (SNPs) associated with blood LDL-C in European ancestry populations (*P* < 5 × 10^–8^) [19]. In our study, among those 11 SNPs reported, four SNPs (rs3846663, rs1501908, rs2650000, rs6102059) were genotyped based on the KoreanChip in the present participants with an MAF above 0.1. Among these four SNPs, rs3846663 is located in HMG-CoA reductase (*HMGCR*), rs1501908 is located between T-cell immunoglobulin and mucin domain-containing protein 4 (*TIMD4*) and hepatitis A virus cellular receptor 1 (*HAVCR1*), rs2650000 is located near hepatocyte nuclear factor 1 homeobox A (*HNF1A*), and rs6102059 is located near MAF BZIP Transcription Factor B (*MAFB*). Detailed information on individual SNPs is shown in Supplementary Table 1.

### Assessment of lifestyle and healt-Hrelated factors

Body weight was measured using a bioelectric impedance analysis machine (InBody 620, Biospace Co. Ltd, Seoul, Korea). Body mass index (BMI) was calculated as weight (kg) divided by height squared (m^2^). Waist circumference and hip circumference were tape-measured, and waist-hip ratio (WHR) was calculated as waist circumference (cm) divided by hip circumference (cm). We obtained the following information from the structured questionnaires: length of stay in Korea, employment status, place of residence, educational level, and vigorous activity. If participants engaged at least one day in vigorous physical activities, which referred to activities that make participants breathe harder than normal, including heavy lifting, digging, aerobics, or fast bicycling, we considered them as vigorously physically active. If participants reported that they were diagnosed by physicians or took medications regarding dyslipidemia, stroke, diabetes, myocardial infarction, heart attack, congestive heart failure, peripheral vascular disease, ischemia, atherosclerosis, and other vascular diseases, they were regarded as having a history of metabolic disorders.

### Statistical analysis

Continuous variables were expressed as means and standard deviation (SD), while categorical variables were expressed as numbers and percentages. Odds ratios (ORs) and corresponding 95% confidence intervals (CIs) for unfavorable lipid profiles were estimated using multivariate logistic regression models. Multivariate models were adjusted for age (years, continuous), total energy intake (kcal/day, continuous), BMI (<18.5, 18.5- <23, 23– <25, ≥25 kg/m^2^), length of stay in Korea (<5, ≥5 years), employment status (employed, unemployed), place of residence in Korea (Seoul, Incheon/Gyeonggido, Daejeon/Chungcheongnamdo), educational levels (elementary or high school, associate or vocational school and college or above), and vigorous activity (no, yes). We additionally adjusted HOMA-IR in the model (HOMA-IR > 2.5 or ≤ 2.5) [20,21] to examine whether the inclusion of insulin resistance variable changed the estimates. In the substitution models, where an increase in dietary fat intake was interpreted as the replacement of carbohydrate with dietary fat, additional macronutrient intakes were included. For example, for energy-adjusted SFA intake as an exposure, the variables included were energy-adjusted protein intake, energy-adjusted MUFA intake, and energy-adjusted PUFA intake in addition to the aforementioned variables. Because the associations were unchanged when we adjusted for alcohol drinking, we did not include alcohol drinking in the models. We did not include smoking status and menopausal status as covariates because most participants were non-smokers and premenopausal women. Missing data (<4% missing) were assigned to median values for continuous variables or the most frequent category for categorical variables. The median fat intake in categories was included as a continuous variable in the model to test for trends. We used restricted cubic splines to examine the possible non-linear associations between dietary fat intake and dyslipidemia when replacing carbohydrate intake, after excluding the highest 1% of SFA intake [22]. To investigate the robustness of the association, we conducted a sensitivity analysis by excluding participants with a history of metabolic disorders (n = 42). We substituted BMI with WHR in the multivariate model to account for abdominal obesity (<0.85, ≥0.85) as a potential confounding factor. We examined whether LDL-C-related SNPs modified the associations by including interaction terms in the models using the likelihood ratio test. All analyses were performed using SAS 9.4 (SAS Institute Inc., Cary, NC, USA). Two-sided *P* < 0.05 were considered statistically significant.

## Results

Among 504 women, we excluded participants if they did not have information on dietary intake (n = 7), had implausible energy intake (3SD above or below the log-transformed mean) (n = 7), or did not have information on blood lipid profiles (n = 12). Women who were pregnant (n = 23) or breastfeeding (n = 49) at recruitment were also excluded. As a result, a total of 406 women were included in the analysis. Participants who self-reported a diagnosis of elevated cholesterol levels or triglycerides (n = 2), or were taking medications for dyslipidemia (n = 1) were additionally excluded when we examined individual lipid biomarkers as outcomes. As a result, a total of 381 women were included in this study. The general characteristics of the study population across tertiles of energy-adjusted intakes of total and specific fats are shown in Table 1. Median fat intakes as a percentage of total energy were 25.56% (20.09–32.89) for total fat, 4.60% (2.35–7.48) for SFAs, 4.95% (2.46–8.13) for MUFAs, and 3.26% (1.48–5.37) for PUFAs. A total of 130 (32.02%) participants had dyslipidemia (high TC, high TG, or high LDL-C). After excluding participants who had been diagnosed with dyslipidemia by physicians, 100 (6.25%) participants had high TC, 36 participants (9.45%) had high TG, 69 participants (18.11%) had high LDL-C, and 106 participants (27.82%) had low HDL-C. Women in the third tertile were relatively younger than those in the other two tertiles. We observed a higher proportion of < 5 years of stay in Korea among Filipino women in the third tertile than among those in the first tertile. Women in the third tertile were more likely to have insulin resistance than those in the first tertile. Body mass index, employment, region of residence, and vigorous activity were similar across fat intakes. Similar baseline characteristics were shown for tertiles of percent energy of total and specific types of fats (Supplementary Table 2).

**Table 1:** Characteristics according to energy-adjusted intakes of total and specific types of fats.


	TOTAL FAT			SATURATED FAT			MONOUNSATURATED FAT			POLYUNSATURATED FAT		
			
	TERTILE 1	TERTILE 2	TERTILE 3	TERTILE 1	TERTILE 2	TERTILE 3	TERTILE 1	TERTILE 2	TERTILE 3	TERTILE 1	TERTILE 2	TERTILE 3

**N**	135	136	135	135	136	135	135	136	135	135	136	135

**Mean (SD)** ^a^												

Age (years)	36.42 (7.83)	35.65 (7.42)	33.44 (8.12)	36.14 (7.83)	35.71 (7.94)	33.66 (7.69)	35.65 (8.09)	36.21 (7.63)	33.64 (7.73)	35.27 (7.74)	35.85 (7.67)	34.39 (8.20)

BMI (kg/m^2^)	23.88 (4.06)	23.91 (3.95)	23.09 (3.41)	24.07 (3.79)	23.36 (3.74)	23.45 (3.94)	23.75 (3.61)	23.65 (4.29)	23.48 (3.57)	23.64 (3.94)	23.96 (4.04)	23.27 (3.47)

Total energy intake (kcal/day)	1786.99 (666.20)	1788.96 (631.23)	1688.62 (667.21)	1783.75 (635.35)	1885.78 (681.78)	1639.33 (627.69)	1918.39 (731.33)	1840.23 (591.71)	1505.57 (559.04)	1862.32 (748.15)	1855.21 (598.72)	1546.55 (557.32)

Carbohydrate (% of total energy)	67.53 (8.96)	57.64 (5.80)	45.51 (8.15)	60.92 (13.09)	58.86 (8.94)	50.89 (10.82)	60.58 (13.28)	58.80 (9.40)	51.30 (10.55)	58.90 (13.78)	58.96 (10.08)	52.82 (10.40)

Protein (% of total energy)	15.15 (5.04)	16.13 (4.09)	16.94 (4.38)	16.28 (5.20)	15.71 (4.04)	16.23 (4.40)	16.29 (5.68)	15.81 (3.74)	16.13 (4.08)	16.18 (5.44)	15.95 (4.25)	16.10 (3.91)

Total fat (% of total energy)	16.99 (4.62)	26.02 (2.81)	37.37 (6.19)	22.22 (9.47)	25.58 (7.18)	32.58 (8.93)	22.65 (9.46)	25.33 (7.40)	32.40 (9.06)	24.08 (10.24)	25.21 (7.99)	31.09 (8.96)

SFA (% of total energy)	3.22 (2.20)	4.95 (3.13)	8.41 (5.37)	1.57 (0.88)	4.58 (1.04)	10.43 (3.85)	2.42 (2.36)	4.75 (2.22)	9.41 (4.64)	3.24 (2.84)	5.63 (4.10)	7.71 (4.75)

MUFA (% of total energy)	3.30 (2.41)	5.44 (3.47)	9.22 (6.18)	2.25 (1.81)	5.22 (2.73)	10.49 (5.33)	1.65 (1.37)	5.09 (1.77)	11.23 (4.67)	2.44 (2.43)	6.15 (4.09)	9.37 (5.23)

PUFA (% of total energy)	2.61 (2.00)	3.76 (2.50)	5.63 (94.59)	2.28 (1.96)	3.89 (3.00)	5.83 (4.08)	1.68 (1.58)	3.84 (2.51)	6.47 (3.95)	1.03 (0.70)	3.33 (1.01)	7.65 (3.42)

**N (%)** ^b^												

Length of Stay in Korea												

< 5years	24 (17.91)	31 (24.03)	50 (38.46)	33 (25.19)	30 (22.90)	42 (32.06)	32 (24.43)	32 (24.43)	41 (31.30)	33 (25.19)	30 (22.22)	42 (33.07)

≥ 5years	110 (82.09)	98 (75.97)	80 (61.54)	98 (74.81)	101 (77.10)	89 (67.94)	99 (75.57)	99 (75.57)	90 (68.70)	98 (74.81)	105 (77.78)	85 (66.93)

Insulin resistance^c^												

No	108 (80.00)	97 (71.32)	100 (74.07)	106 (78.52)	106 (77.94)	93 (68.89)	106 (78.52)	107 (78.68)	92 (68.15)	105 (77.78)	106 (77.94)	94 (69.63)

Yes	27 (20.00)	39 (28.68)	35 (25.93)	29 (21.48)	30 (22.06)	42 (31.11)	29 (21.48)	29 (21.32)	43 (31.85)	30 (22.22)	30 (22.06)	41 (30.37)

Employment status												

Employed	59 (43.7)	61 (45.19)	59 (44.03)	55 (40.74)	67 (50.00)	57 (42.22)	58 (43.28)	58 (42.96)	63 (46.67)	53 (39.85)	69 (50.74)	57 (42.22)

Unemployed	76 (56.3)	74 (54.81)	75 (55.97)	80 (59.26)	67 (50.00)	78 (57.78)	76 (56.72)	77 (57.04)	72 (53.33)	80 (60.15)	67 (49.26)	78 (57.78)


*Abbreviations:* BMI, body mass index; SFA, saturated fat; MUFA, monounsaturated fat; PUFA, polyunsaturated fat. There were missing data for a few participants; BMI (n = 2), length of stay in Korea (n = 13), and occupation (n = 2). ^a^ Mean (SD) for continuous variables ^b^ n (%) for categorical variables. ^c^ Insulin resistance was defined as HOMA-IR above 2.5.

We found that SFA intake was associated with a high prevalence of dyslipidemia and elevated levels of TC and LDL-C (Table 2). When carbohydrate intake was replaced with SFA intake, the ORs (95% CIs) for dyslipidemia were 2.28 (1.19–4.35) for the second tertile and 2.88 (1.29–6.39; *P for trend* = 0.02) for the third tertile compared with the first tertile. Additional adjustment for insulin resistance slightly attenuated the associations; ORs (95% CIs) were 2.28 (1.18–4.41) for the second tertile and 2.65 (1.18–5.93) for the third tertile of SFA intake (*P for trend* = 0.03). Replacing 10 g/day of SFA with an equivalent energy intake from carbohydrates was associated with a 1.68 fold (95% CI = 1.11–2.55) higher prevalence of dyslipidemia. ORs (95% CIs) for the third tertile compared with the first tertile of SFA intake were 3.62 (1.53–8.55; *P for trend* = 0.01) for elevated TC and 4.00 (1.48–10.79; *P for trend* = 0.02) for elevated LDL-C. Replacement of carbohydrate with a 10 g/day increment of SFAs was associated with a high prevalence of elevated TC.

**Table 2:** Odds ratio (OR)s and 95% confidence interval (CI)s for dyslipidemia according to dietary saturated fat (SFA) replacing carbohydrate intake.


	TERTILES OF ENERGY-ADJUSTED DIETARY SFA INTAKE				

	TERTILE 1	TERTILE 2	TERTILE 3	*P FOR TREND*	PER 10 G OF SFA INTAKE^e^

**Median intake^a^, g/day**	3.37	8.96	18.39		

**Dyslipidemia**					

No. of cases/total	38/135	49/136	43/135		

Age- and energy-adjusted model^b^	1.00	1.94 (1.05–3.57)	2.36 (1.11–5.05)	0.04	1.50 (1.02–2.21)

Multivariate model 1^c, f^	1.00	2.28 (1.19–4.35)	2.88 (1.29–6.39)	0.02	1.68 (1.11–2.55)

Multivariate model 2^d^	1.00	2.28 (1.18–4.41)	2.65 (1.18–5.93)	0.03	1.64 (1.07–2.52)

**TC ≥ 200 mg/dL**					

No. of cases/total	25/127	38/127	37/127		

Age- and energy-adjusted model^b^	1.00	2.30 (1.16–4.55)	2.96 (1.29–6.79)	0.02	1.47 (0.98–2.22)

Multivariate model 1^c, f^	1.00	2.76 (1.34–5.66)	3.62 (1.53–8.55)	0.01	1.58 (1.03–2.43)

Multivariate model 2^d^	1.00	2.69 (1.31–5.53)	3.34 (1.41–7.90)	0.02	1.58 (1.03–2.43)

**TG ≥ 150 mg/dL**					

No. of cases/total	11/127	16/127	9/127		

Age- and energy-adjusted model	1.00	1.80 (0.70–4.64)	1.16 (0.33–4.08)	0.99	0.78 (0.39–1.54)

Multivariate model 1^c, f^	1.00	2.07 (0.77–5.61)	1.46 (0.42–5.10)	0.72	0.87 (0.43–1.76)

Multivariate model 2^d^	1.00	1.71 (0.59–4.95)	1.08 (0.28–4.20)	0.96	0.93 (0.45–1.90)

**LDL-C ≥ 130 mg/dL**					

No. of cases/total	17/127	26/127	26/127		

Age- and energy-adjusted model^b^	1.00	2.21 (1.01–4.80)	2.93 (1.14–7.51)	0.04	1.43 (0.91–2.26)

Multivariate model 1^c, f^	1.00	3.02 (1.32–6.91)	4.00 (1.48–10.79)	0.02	1.55 (0.94–2.53)

Multivariate model 2^d^	1.00	2.99 (1.29–6.90)	3.71 (1.37–10.05)	0.02	1.58 (0.97–2.58)

**HDL-C < 50 mg/dL**					

No. of cases/total	39/127	34/127	33/127		

Age- and energy-adjusted model^b^	1.00	0.76 (0.40–1.43)	0.61 (0.28–1.34)	0.24	0.86 (0.57–1.28)

Multivariate model 1^c, f^	1.00	0.93 (0.47–1.83)	0.69 (0.30–1.59)	0.36	0.93 (0.60–1.43)

Multivariate model 2^d^	1.00	0.82 (0.41–1.65)	0.57 (0.24–1.34)	0.19	0.90 (0.58–1.39)


*Abbreviations:* SFA, saturated fat; TC, total cholesterol; TG, triglyceride; LDL-C, LDL cholesterol; HDL-C, HDL cholesterol. Dyslipidemia was defined as total cholesterol ≥200 mg/dL or triglyceride ≥150 mg/dL or LDL cholesterol ≥130 mg/dL. Estimates are presented as odds ratio (OR)s and 95% confidence interval (CI)s. ^a^ Median (g/day) of energy-adjusted SFA intakes were calculated. ^b^ Model was adjusted for age (years, continuous), total energy intake (kcal/day, continuous), energy-adjusted protein (tertile), and energy-adjusted other types of fats (tertile). ^c^ Model was adjusted for age (years, continuous), total energy intake (kcal/day, continuous), BMI (<18.5, 18.5– <23, 23- <25, ≥25 kg/m^2^), length of stay in Korea (<5 years, ≥5 years), employment status (employed, unemployed), region (Seoul, Incheon/Gyeonggido, Daejeon/Chuncheongnamdo), vigorous activity (no, yes), educational level (elementary or high school, association or vocational or college or above), energy-adjusted protein (tertile), and energy-adjusted other types of fats (tertile). ^d^ Model was additionally adjusted for insulin resistance (no: HOMA-IR < 2.5, yes: HOMA-IR ≥ 2.5). ^e^ For 10 g/day increment of SFA. Energy-adjusted protein and other types of fats were included in the model as continuous variables (g/day). ^f^ The results remained similar when waist to hip circumference was adjusted instead of BMI in the multivariate model 1^c^ (Supplementary Table 6).

Replacing carbohydrate intake with MUFA intake showed an inverse association (Table 3). ORs (95% CIs) of dyslipidemia for subsequent tertiles compared with the first tertile were 0.66 (0.33–1.34) and 0.36 (0.14–0.92), respectively (*P for trend* = 0.03). The association persisted when we additionally adjusted for insulin resistance (*P for trend* = 0.02). The associations of PUFAs and total fat with dyslipidemia were not statistically significant (Tables 4 and 5). For specific lipid biomarkers, there were tendencies of elevated TG and LDL-C with total fat and of low TG and LDL-C with MUFA intake.

**Table 3:** Odds ratio (OR)s and 95% confidence interval (CI)s for dyslipidemia according to dietary monounsaturated fat (MUFA) replacing carbohydrate intake.


	TERTILES OF ENERGY-ADJUSTED DIETARY MUFA INTAKE				

	TERTILE 1	TERTILE 2	TERTILE 3	*P FOR TREND*	PER 10 G OF MUFA INTAKE^e^

**Median intake^a^, g/day**	2.70	9.55	23.37		

**Dyslipidemia**					

No. of cases/total	46/135	46/136	38/135		

Age- and energy-adjusted model^b^	1.00	0.68 (0.35–1.33)	0.45 (0.18–1.11)	0.09	0.81 (0.59–1.11)

Multivariate model 1^c, f^	1.00	0.66 (0.33–1.34)	0.36 (0.14–0.92)	0.03	0.73 (0.52–1.03)

Multivariate model 2^d^	1.00	0.66 (0.32–1.35)	0.33 (0.13–0.88)	0.02	0.71 (0.49–1.02)

**TC ≥ 200 mg/dL**					

No. of cases/total	33/127	33/127	34/127		

Age- and energy-adjusted model^b^	1.00	0.68 (0.32–1.43)	0.62 (0.22–1.73)	0.49	0.93 (0.67–1.29)

Multivariate model 1^c, f^	1.00	0.65 (0.30–1.42)	0.52 (0.18–1.50)	0.30	0.88 (0.62–1.26)

Multivariate model 2^d^	1.00	0.67 (0.31–1.47)	0.56 (0.19–1.61)	0.34	0.87 (0.60–1.24)

**TG ≥ 150 mg/dL**					

No. of cases/total	14/127	11/127	11/127		

Age- and energy-adjusted model^b^	1.00	0.41 (0.14–1.24)	0.35 (0.08–1.56)	0.29	1.24 (0.78–1.97)

Multivariate model 1^c, f^	1.00	0.35 (0.11–1.10)	0.26 (0.06–1.16)	0.15	1.19 (0.72–1.99)

Multivariate model 2^d^	1.00	0.32 (0.09–1.09)	0.20 (0.04–1.09)	0.11	1.04 (0.60–1.82)

**LDL-C ≥ 130 mg/dL**					

No. of cases/total	24/127	21/127	24/127		

Age- and energy-adjusted model^b^	1.00	0.64 (0.27–1.49)	0.72 (0.23–2.28)	0.80	1.00 (0.70–1.44)

Multivariate model 1^c, f^	1.00	0.51 (0.21–1.24)	0.54 (0.16–1.77)	0.52	0.97 (0.66–1.44)

Multivariate model 2^d^	1.00	0.50 (0.20–1.24)	0.57 (0.17–1.91)	0.60	0.93 (0.62–1.40)

**HDL-C < 50 mg/dL**					

No. of cases/total	38/127	31/127	37/127		

Age- and energy-adjusted model^b^	1.00	0.90 (0.44–1.82)	1.24 (0.46–3.33)	0.53	1.19 (0.87–1.62)

Multivariate model 1^c, f^	1.00	0.83 (0.39–1.76)	1.10 (0.39–3.12)	0.69	1.11 (0.80–1.54)

Multivariate model 2^d^	1.00	0.86 (0.40–1.84)	1.19 (0.41–3.47)	0.61	1.09 (0.78–1.53)


*Abbreviations:* MUFA, monounsaturated fat; TC, total cholesterol; TG, triglyceride; LDL-C, LDL cholesterol; HDL-C, HDL cholesterol. Dyslipidemia was defined as total cholesterol ≥200 mg/dL or triglyceride ≥150 mg/dL or LDL cholesterol ≥130 mg/dL. Estimates are presented as odds ratio (OR)s and 95% confidence interval (CI)s. ^a^ Median (g/day) of energy-adjusted MUFA intakes were calculated. ^b^ Model was adjusted for age (years, continuous), total energy intake (kcal/day, continuous), energy-adjusted protein (tertile), and energy-adjusted other types of fats (tertile). ^c^ Model was adjusted for age (years, continuous), total energy intake (kcal/day, continuous), BMI (<18.5, 18.5- <23, 23- <25, ≥25 kg/m^2^), length of stay in Korea (<5 years, ≥5 years), employment status (employed, unemployed), region (Seoul, Incheon/Gyeonggido, Daejeon/Chuncheongnamdo), vigorous activity (no, yes), educational level (elementary or high school, association or vocational or college or above), energy-adjusted protein (tertile), and energy-adjusted other types of fats (tertile). ^d^ Model was additionally adjusted for insulin resistance (no: HOMA-IR <2.5, yes: HOMA-IR ≥2.5). ^e^ For 10 g/day increment of MUFA. Energy-adjusted protein and other types of fats were included in the model as continuous variables (g/day). ^f^ The results remained similar when waist to hip circumference was adjusted instead of BMI in the multivariate model 1^c^ (Supplementary Table 6).

**Table 4:** Odds ratio (OR)s and 95% confidence interval (CI)s for dyslipidemia according to dietary polyunsaturated fat (PUFA) replacing carbohydrate intake.


	TERTILES OF ENERGY-ADJUSTED DIETARY PUFA INTAKE				

	TERTILE 1	TERTILE 2	TERTILE 3	*P FOR TREND*	PER 10 G OF PUFA INTAKE^e^

**Median intake^a^, g/day**	2.03	6.26	14.22		

**Dyslipidemia**					

No. of cases/total	46/135	41/136	43/135		

Age- and energy-adjusted model^b^	1.00	0.85 (0.46–1.56)	1.15 (0.57–2.33)	0.53	1.08 (0.77–1.52)

Multivariate model 1^c, f^	1.00	0.84 (0.43–1.62)	1.24 (0.58–2.65)	0.43	1.18 (0.81–1.71)

Multivariate model 2^d^	1.00	0.87 (0.44–1.71)	1.18 (0.54–2.58)	0.56	1.16 (0.78–1.71)

**TC ≥ 200 mg/dL**					

No. of cases/total	34/127	31/127	35/127		

Age- and energy-adjusted model^b^	1.00	0.85 (0.43–1.69)	1.02 (0.46–2.29)	0.80	1.11 (0.78–1.58)

Multivariate model 1^c, f^	1.00	0.78 (0.37–1.64)	1.03 (0.44–2.43)	0.72	1.20 (0.82–1.76)

Multivariate model 2^d^	1.00	0.79 (0.38–1.67)	0.97 (0.41–2.30)	0.88	1.17 (0.79–1.74)

**TG ≥ 150 mg/dL**					

No. of cases/total	11/127	10/127	15/127		

Age- and energy-adjusted model^b^	1.00	1.36 (0.48–3.83)	2.93 (0.88–9.75)	0.06	0.97 (0.58–1.64)

Multivariate model 1^c, f^	1.00	1.39 (0.47–4.13)	3.17 (0.91–11.10)	0.05	0.99 (0.55–1.79)

Multivariate model 2^d^	1.00	1.68 (0.53–5.33)	3.43 (0.84–13.97)	0.08	0.88 (0.44–1.77)

**LDL-C ≥ 130 mg/dL**					

No. of cases/total	25/127	21/127	23/127		

Age- and energy-adjusted model^b^	1.00	0.75 (0.34–1.65)	0.80 (0.32–2.01)	0.77	1.01 (0.67–1.50)

Multivariate model 1^c, f^	1.00	0.88 (0.38–2.03)	0.94 (0.35–2.51)	0.98	1.11 (0.71–1.73)

Multivariate model 2^d^	1.00	0.90 (0.38–2.11)	0.81 (0.29–2.23)	0.69	1.05 (0.65–1.70)

**HDL-C <50 mg/dL**					

No. of cases/total	37/127	32/127	37/127		

Age- and energy-adjusted model^b^	1.00	0.87 (0.46–1.66)	1.06 (0.49–2.28)	0.76	0.81 (0.56–1.16)

Multivariate model 1^c, f^	1.00	0.88 (0.44–1.74)	1.08 (0.48–2.44)	0.73	0.84 (0.57–1.23)

Multivariate model 2^d^	1.00	0.90 (0.44–1.81)	0.97 (0.41–2.28)	0.99	0.78 (0.51–1.17)


*Abbreviations:* PUFA, polyunsaturated fat; TC, total cholesterol; TG, triglyceride; LDL-C, LDL cholesterol; HDL-C, HDL cholesterol. Dyslipidemia was defined as total cholesterol ≥200 mg/dL or triglyceride ≥150 mg/dL or LDL cholesterol ≥130 mg/dL. Estimates are presented as odds ratio (OR)s and 95% confidence interval (CI)s. ^a^ Median (g/day) of energy-adjusted PUFA intakes were calculated. ^b^ Model was adjusted for age (years, continuous), total energy intake (kcal/day, continuous), energy-adjusted protein (tertile), and energy-adjusted other types of fats (tertile). ^c^ Model was adjusted for age (years, continuous), total energy intake (kcal/day, continuous), BMI (<18.5, 18.5- <23, 23- <25, ≥25 kg/m^2^), length of stay in Korea (<5 years, ≥5 years), employment status (employed, unemployed), region (Seoul, Incheon/Gyeonggido, Daejeon/Chuncheongnamdo), vigorous activity (no, yes), educational level (elementary or high school, association or vocational or college or above), energy-adjusted protein (tertile), and energy-adjusted other types of fats (tertile). ^d^ Model was additionally adjusted for insulin resistance (no: HOMA-IR <2.5, yes: HOMA-IR ≥2.5). ^e^ For 10 g/day increment of PUFA. Energy-adjusted protein and other types of fats were included in the model as continuous variables (g/day). ^f^ The results remained similar when waist to hip circumference was adjusted instead of BMI in the multivariate model 1^c^ (Supplementary Table 6).

**Table 5:** Odds ratio (OR)s and 95% confidence interval (CI)s for dyslipidemia according to dietary total fat replacing carbohydrate intake.


	TERTILES OF ENERGY-ADJUSTED DIETARY TOTAL FAT INTAKE				

	TERTILE 1	TERTILE 2	TERTILE 3	*P FOR TREND*	PER 10 G OF TOTAL FAT INTAKE^e^

**Median intake^a^, g/day**	35.09	50.27	72.70		

**Dyslipidemia**					

No. of cases/total	43/135	47/136	40/135		

Age- and energy-adjusted model^b^	1.00	1.21 (0.71–2.04)	1.13 (0.65–1.95)	0.71	1.07 (0.95–1.20)

Multivariate model 1^c, f^	1.00	1.21 (0.69–2.1)	1.22 (0.68–2.18)	0.52	1.10 (0.97–1.25)

Multivariate model 2^d^	1.00	1.07 (0.6–1.89)	1.14 (0.63–2.06)	0.68	1.09 (0.96–1.25)

**TC ≥ 200 mg/dL**					

No. of cases/total	33/127	36/127	31/127		

Age- and energy-adjusted model^b^	1.00	1.15 (0.65–2.04)	1.13 (0.62–2.06)	0.72	1.09 (0.96–1.25)

Multivariate model 1^c, f^	1.00	1.12 (0.61–2.03)	1.26 (0.67–2.36)	0.48	1.14 (0.99–1.31)

Multivariate model 2^d^	1.00	1.05 (0.57–1.92)	1.20 (0.63–2.27)	0.57	1.13 (0.98–1.30)

**TG ≥ 150 mg/dL**					

No. of cases/total	12/127	13/127	11/127		

Age- and energy-adjusted model^b^	1.00	1.21 (0.52–2.82)	1.19 (0.48–2.94)	0.72	1.02 (0.84–1.23)

Multivariate model 1^c, f^	1.00	1.23 (0.51–2.94)	1.51 (0.59–3.84)	0.39	1.08 (0.88–1.33)

Multivariate model 2^d^	1.00	0.95(0.37–2.46)	1.26 (0.47–3.42)	0.63	1.04 (0.83–1.30)

**LDL-C ≥ 130 mg/dL**					

No. of cases/total	20/127	25/127	24/127		

Age- and energy-adjusted model^b^	1.00	1.38 (0.71–2.69)	1.61 (0.80–3.21)	0.20	1.12 (0.97–1.30)

Multivariate model 1^c, f^	1.00	1.43 (0.71–2.88)	1.84 (0.89–3.84)	0.11	1.20 (1.02–1.40)

Multivariate model 2^d^	1.00	1.30 (0.63–2.65)	1.71 (0.81–3.60)	0.16	1.18 (1.001–1.39)

**HDL-C < 50 mg/dL**					

No. of cases/total	38/127	36/127	32/127		

Age- and energy-adjusted model^b^	1.00	0.95 (0.55–1.65)	0.76 (0.43–1.35)	0.33	0.90 (0.79–1.02)

Multivariate model 1^c, f^	1.00	1.04 (0.58–1.85)	0.83 (0.44–1.53)	0.52	0.92 (0.80–1.05)

Multivariate model 2^d^	1.00	0.92 (0.50–1.67)	0.73 (0.38–1.39)	0.33	0.89 (0.77–1.03)


*Abbreviations:* TC, total cholesterol; TG, triglyceride; LDL-C, LDL cholesterol; HDL-C, HDL cholesterol. Dyslipidemia was defined as total cholesterol ≥200 mg/dL or triglyceride ≥150 mg/dL or LDL cholesterol ≥130 mg/dL. Estimates are presented as odds ratio (OR)s and 95% confidence interval (CI)s. ^a^ Median (g/day) of energy-adjusted total fat intakes were calculated. ^b^ Model was adjusted for age (years, continuous), total energy intake (kcal/day, continuous), and energy-adjusted protein (tertile). ^c^ Model was adjusted for age (years, continuous), total energy intake (kcal/day, continuous), BMI (<18.5, 18.5- <23, 23- <25, ≥25 kg/m^2^), length of stay in Korea (<5 years, ≥5 years), employment status (employed, unemployed), region (Seoul, Incheon/Gyeonggido, Daejeon/Chuncheongnamdo), vigorous activity (no, yes), educational level (elementary or high school, association or vocational or college or above), and energy-adjusted protein (tertile). ^d^ Model was additionally adjusted for insulin resistance (no: HOMA-IR <2.5, yes: HOMA-IR ≥2.5). ^e^ For 10 g/day increment of total fat. Energy-adjusted protein were included in the model as continuous variables (g/day). ^f^ The results remained similar when waist to hip circumference was adjusted instead of BMI in the multivariate model 1^c^ (Supplementary Table 6).

**Figure 1:**
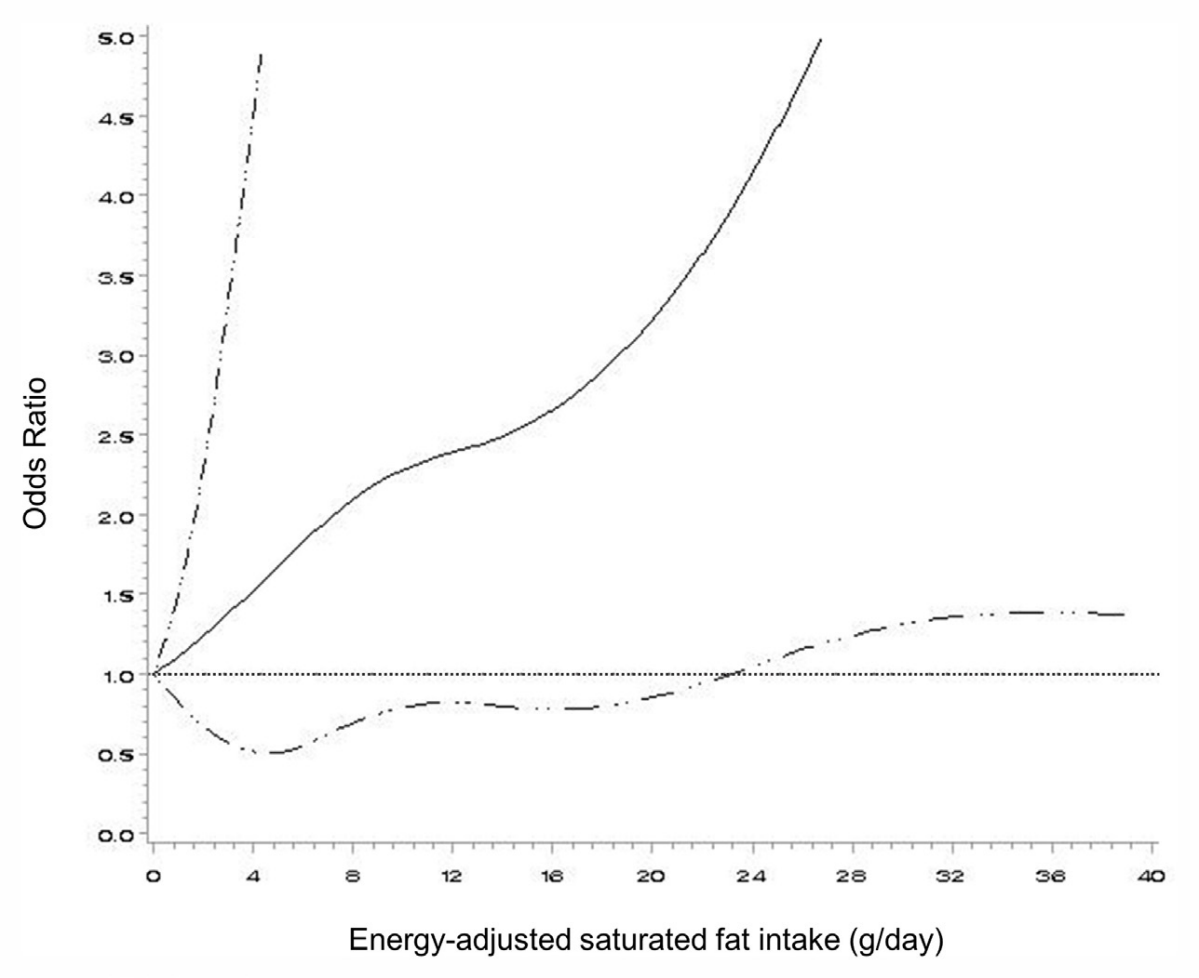
Restricted cubic spline models for dyslipidemia according to energy-adjusted SFA replacing carbohydrate intake. Models were adjusted for age (years, continuous), total energy intake (kcal/day, continuous), BMI (<18.5, 18.5- <23, 23– <25, ≥25 kg/m^2^), length of stay in Korea (<5 years, ≥5 years), employment status (employed, unemployed), region (Seoul, Incheon/Gyeonggido, Daejeon/Chuncheongnamdo), vigorous activity (no, yes), educational level (elementary or high school, association or vocational or college or above), energy-adjusted protein (g/day, continuous), energy-adjusted MUFA intake (g/day, continuous), and energy-adjusted PUFA (g/day, continuous), with five knots (*P for curvature* = 0.92, *P for linearity* = 0.02). A solid line indicates the odds ratio, and the dashed double-dotted line indicates 95% confidence intervals.

The associations for dietary fat intake were also examined using a standard multivariate model including energy intake but not protein or other types of fat (no substitution model). Dietary SFA intake was associated with a high prevalence of TC levels ≥ 200 mg/dL or LDL-C levels ≥ 130 mg/dL (Supplementary Table 3). ORs (95% CIs) for the third tertile compared with those for the first tertile of SFA intake were 2.35 (1.25–4.43; *P for trend* = 0.01) for TC and 2.52 (1.21–5.26; *P for trend* = 0.02) for LDL-C. No clear associations were found for total fat, MUFA or PUFA intake. When an equivalent energy intake from carbohydrate replaced with percent energy intake from total and specific types of fats, the associations for SFAs and MUFAs became more apparent (Supplementary Table 4). Women in the third tertile had a 3.69 times (1.57–8.68) higher prevalence of dyslipidemia than those in the first tertile (*P for trend* = 0.004). For a 5% increment in energy intake from SFAs replacing carbohydrate intake, the OR (95% CI) was 2.12 (1.28–3.53). The third tertile of percent energy intake from SFA was associated with a 5.23 times higher prevalence of TC levels ≥ 200 mg/dL and a 5.60 times higher prevalence of LDL-C levels ≥ 130 mg/dL. Additionally, a lower prevalence of HDL-C levels < 50 mg/dL was observed for percent energy intake from SFA. When carbohydrates were isocalorically replaced with MUFAs, ORs (95% CIs) for the third tertile compared with those for the first tertile were 0.28 (0.11–0.72; *P for trend* = 0.01) for dyslipidemia and 0.26 (0.09–0.75; *P for trend* = 0.01) for TC levels ≥ 200 mg/dL. Replacement of 5% of energy from carbohydrate with energy from MUFA resulted in an OR (95% CI) of 0.55 (0.33–0.91) for dyslipidemia. In sensitivity analysis, in which we excluded individuals with a history of metabolic disorder, the associations for dietary fat intake as a replacement for carbohydrate intake were similar (Supplementary Table 5). Substituting BMI with WHR in the regression models has shown similar results, albeit with a slight attenuation (Supplementary Table 6). When we examined the possible non-linear association between SFA intake and dyslipidemia in the restricted cubic spline, we observed a linear association (*P for curvature* = 0.92, *P for linearity* = 0.02) (Figure 1).

### Subgroup analyses of genetic polymorphisms

We examined whether the association between replacement of carbohydrate intake with SFA and dyslipidemia differed by genetic polymorphisms related to LDL-C levels (Table 6). We did not observe significant interactions by genetic polymorphisms of rs3846663, rs1501908, or rs2650000. However, for every 10 g/day increment in energy-adjusted SFA intake, the ORs (95% CIs) for dyslipidemia were 1.61 (0.59–4.43) among participants with CC genotypes of rs6102059 and 1.35 (0.80–2.28) among those with TT + TC genotypes (*P for interaction* = 0.01). The associations between energy-adjusted SFA intake and LDL-hypercholesterolemia did not vary according to LDL-C-related polymorphisms of rs3846663, rs1501908, rs2650000, or rs6102059 (*P for interaction* = 0.75, 0.67, 0.89, and 0.28, respectively). Among participants with the minor alleles of SNPs, ORs (95% CI) for 10 g/day increment in energy-adjusted SFA intake in relation to elevated LDL-C were 1.92 (1.03–3.58), 1.44 (0.68–3.05), 1.68 (0.90–3.14), and 1.58 (0.82–3.03) for rs3846663, rs1501908, rs2650000, and rs6102059, respectively. The associations were more pronounced in the presence of minor alleles, although the interactions did not reach statistical significance.

**Table 6:** Subgroup analysis by genetic polymorphisms related to LDL cholesterol according to SFA intake replacing carbohydrate intake.


	ENERGY ADJUSTED DIETARY SFA INTAKE (PER 10 G INCREMENT/DAY)	

	OR (95% CI)^c^	*P FOR INTERACTION*

**Dyslipidemia** ^a^		

**rs3846663 (T < C)**	n = 374	0.43

**CC**	1.89 (0.84–4.23)	

**TT + TC**	1.49 (0.87–2.57)	

**rs1501908 (G < C)**	n = 374	0.65

**CC**	1.28 (0.64–2.57)	

**GG + GC**	1.94 (1.02–3.68)	

**rs2650000 (A < C)**	n = 374	0.96

**CC**	1.68 (0.68–4.13)	

**AA + AC**	1.56 (0.93–2.62)	

**rs6102059 (T < C)**	n = 372	0.01

**CC**	1.61 (0.59–4.43)	

**TT + TC**	1.35 (0.80–2.28)	

**LDL-C ≥ 130 mg/dL**		

**rs3846663 (T < C)**	n = 352	0.75

**CC**	0.75 (0.22–2.56)	

**TT + TC**	1.92 (1.03–3.58)	

**rs1501908 (G < C)**	n = 352	0.67

**CC**	1.83 (0.80–4.19)	

**GG + GC**	1.44 (0.68–3.05)	

**rs2650000 (A < C)**	n = 352	0.89

**CC**	1.44 (0.52–4.02)	

**AA+AC**	1.68 (0.90–3.14)	

**rs6102059 (T < C)**	n = 350	0.28

**CC**	1.64 (0.52–5.19)	

**TT + TC**	1.58 (0.82–3.03)	


*Abbreviations:* SFA, saturated fat; LDL-C, LDL cholesterol. ^a^ Dyslipidemia: total cholesterol ≥200 mg/dL or triglyceride ≥150 mg/dL or LDL-C ≥130 mg/dL. ^c^ Estimates are presented as odds ratios (OR)s and 95% confidence interval (CI)s. Models were adjusted for age (years, continuous), energy-adjusted protein (g/day, continuous), energy-adjusted MUFA (g/day, continuous), energy-adjusted PUFA (g/day, continuous), total energy intake (kcal/day, continuous), BMI (<18.5, 18.5- <23, 23- <25, ≥25 kg/m^2^), length of stay in Korea (<5 years, ≥5 years), employment status (employed, unemployed), region (Seoul, Incheon/Gyeonggido, Daejeon/Chuncheongnamdo), vigorous activity (no, yes), and educational level (elementary or high school, association or vocational or college or above).

### Foods contributing to dietary fat intake

When we identified the top five foods contributing to the intake of total and specific types of fat, the top contributing food of total fat, SFA, and MUFA was pork; total fat (17.97%), SFA (39.05%), and MUFA (38.67%) (Table 7). Egg (8.42%), milk (7.22%), soybean oil (6.11%), and mackerel (3.76%) contributed to dietary SFA intake. Regarding PUFAs, soybean oil contributed the most (34.79%), followed by pork (13.74%), sesame oil (10.05%), corn oil (6.01%), and egg (5.90%).

**Table 7:** Top five foods contributing to dietary total fat and specific types of fat intake.


RANKING	TOTAL FAT	SFA	MUFA	PUFA

1	Pork (17.97%)	Pork (39.05%)	Pork (38.67%)	Soybean oil (34.79%)

2	Chicken (9.31%)	Egg (8.42%)	Egg (10.60%)	Pork (13.74%)

3	Soybean oil (8.83%)	Milk (7.22%)	Soybean oil (9.24%)	Sesame oil (10.05%)

4	Egg (4.71%)	Soybean oil (6.11%)	Sesame oil (5.74%)	Corn oil (6.01%)

5	Beef (4.63%)	Mackerel (3.76%)	Mackerel (4.69%)	Egg (5.90%)

Cumulative %	45.46%	64.54%	68.93%	70.50%


*Abbreviations:* SFA, saturated fat; MUFA, monounsaturated fat; PUFA, polyunsaturated fat.

## Discussion

We found that dietary SFA intake was associated with a high prevalence of dyslipidemia, elevated TC, and elevated LDL-C, especially when carbohydrates were replaced with SFAs, in Filipino women residing in Korea. Compared with the first tertile of SFA intake (approximately 3 g/day of median intake), the prevalence values of elevated TC and elevated LDL-C were 3.62 and 4.00 times higher in the third tertile (approximately 18 g/day of median SFA intake), respectively. Replacing a 10 g/day SFA intake for an equivalent energy intake from carbohydrates increased the prevalence of dyslipidemia by 68%. The associations of dietary SFA intake with dyslipidemia, elevated TC and elevated LDL-C were similar when percent energy from SFA intake was analyzed. Dietary MUFA intake showed a trend toward decreased prevalence of dyslipidemia. Additionally, dietary intake of total fat or PUFAs was not associated with dyslipidemia. The association between SFA intake and prevalence of dyslipidemia was more pronounced among Filipino women with homozygous major allele carriers of rs6102059 near *MAFB*.

Our findings agree with a previous meta-analysis that an increase in SFA intake increases circulating levels of LDL-C and TC when substituting carbohydrates [23]. A meta-analysis of randomized controlled trials reported that a reduction in SFA intake led to a decrease in the serum levels of TC and LDL-C [24]. Studies included in these meta-analyses were primarily conducted in North America and Europe. The Shanghai Diet and Health Survey (SDHS) observed no association between dietary SFA and unfavorable lipid profiles in Chinese women [25]. In a Japanese cross-sectional study, women with high SFA intake were likely to have higher serum TC and LDL-C [26]. Meanwhile, the INTERLIPID Study, another Japanese cross-sectional study, found that SFA intake was not associated with circulating levels of LDL-C and TC [27]. A Korean cross-sectional study found that the prevalence of hypercholesterolemia and LDL-hypercholesterolemia were 25% and 37% higher in the third tertile, respectively, than in the first tertile of dietary SFA intake [28]. A few randomized trials in Europe reported that SFA-rich diets increased cholesterol levels of LDL fraction [29], while PUFA intake lowered fasting TC levels [30,31].

Previous meta-analyses have shown that increasing PUFA intake and substituting it for carbohydrates reduced circulating levels of LDL-C, TC, or TG [32]. However, we did not find significant inverse associations between PUFA intake and unfavorable lipid profiles. Shanghai Diet and Health Survey reported that neither dietary PUFA nor MUFA intake were associated with dyslipidemia [25]. While our findings suggest that MUFA intake may be associated with a lower prevalence of dyslipidemia when dietary MUFA intake replaced carbohydrates. A meta-analysis of randomized clinical trials reported that MUFA-rich canola oils reduced circulating levels of TC and LDL-C compared to SFA or sunflower oils [33] and levels of TG in comparison to high carbohydrate diets among diabetic patients [34]. Meta-analyses have shown that a low-fat diet may lower plasma TC and LDL-C levels, while a high-fat diet may lower TG [35,36]. In the China Nutrition and Health Surveillance (CNHS) (2015–2017), dietary total fat was associated with increased levels of TC, LDL-C, and HDL-C, although stronger associations were observed for dietary SFA [37]. However, we did not find significant associations between total fat and unfavorable lipid profile.

There are a few potential mechanisms underlying unfavorable lipid responses to high dietary SFA intake. One possible explanation is an accumulation of circulating free fatty acids. The low affinity of SFA to peroxisome proliferator-activated receptor γ (PPARγ) [38] may delay the uptake of circulating free fatty acids, possibly through reduced upregulation of fatty acid transporters [39,40]. The accumulation of free fatty acids enhances the secretion of very low density lipoprotein (VLDL) [41], which can be further metabolized to LDL [42]. Mechanisms related to scavenger receptor class B type 1 (SRB1) may partly explain the increased LDL-C with SFA intake. SRB1 functions as an HDL receptor, but it also uptakes circulating LDL-C [43]. Compared with PUFAs, SFAs downregulate SRB1 [44], which may hinder the clearance of LDL-C. Dietary fat intake may affect the transcriptional and translational levels of genes involved in cholesterol metabolism. A high SFA meal increased the ATP binding cassette transporter A1 (*ABCA1*) and ATP binding cassette transporter G1 (*ABCG1*) gene expression, one of the key mediators of cholesterol efflux in macrophages, compared with an n-6 PUFA rich meal [45,46]. A randomized controlled trial has shown reduced expression levels of LDL receptor (*LDLR*) mRNA with increasing dietary SFA intake compared with n-6 PUFA intake [45] and increased blood serum levels of LDLR protein with decreasing SFA intake [47], supporting evidence of the transcriptional and translational regulation of LDLR as an underlying mechanism. Inhibiting the synthesis of VLDL-C and apolipoproteins could be a potential mechanism by which PUFA reduces circulating TG levels [48]. MUFA-rich diets may reduce serum TG levels by upregulating PPAR family genes [49] and inhibiting hepatic lipogenesis [50], which leads to decreased synthesis of TG and TC.

Genetic predisposition may be associated with susceptibility to dyslipidemia with dietary fat intake. Potential associations of polymorphisms of genes related to lipid metabolism and signaling, including fatty acid desaturase 2 (*FADS2*), cholesteryl ester transfer protein (*CETP*) Taq1B, apolipoprotein A5 (*APOA5*), and leptin receptor (*LEPR*), with the level of blood cholesterols have been reported [51,52,53]. Meta-analyses of the European ancestry genome-wide association study (GWAS) suggested that variants of rs3846663 in *HMGCR*, rs1501908 between *TIMD4* and *HAVCR1*, rs2650000 near *HNF1A*, and rs6102059 near *MAFB* were associated with dyslipidemia [19]. Among them, the presence of minor alleles of rs1501908 and rs6102059 was inversely associated with blood LDL-C levels [19]. In our study, the association between SFA intake and the prevalence of dyslipidemia varied by genetic polymorphisms of rs6102059 near *MAFB*. MAFB affected *LDLR* mRNA expression by regulating hairy and enhancer of split 1 (*HES1*) and neurogenic locus notch homolog protein 2 (*NOTCH2*) [54,55] and stimulated cholesterol efflux in macrophages by upregulating *Abca1* and *Abcg1* expression in a mouse model [56]. Our study findings of effect modification by polymorphisms related to LDL-C level warrant further replications.

The mean intake of dietary SFAs was approximately 10 g per day among middle-aged Korean women from 2013 to 2015 [10], which was similar to the intake of Filipino women in our study (mean ± SD = 10.75 ± 9.76 g). Major foods contributing to dietary SFA intake in Korea were pork, beef, milk, egg, ramyun (instant noodles), mixed coffee, and bread [57]. This result was comparable with contributing foods in our study.

To our best knowledge, this study is the first to examine the associations between dietary fat intake and the blood lipid profile among Filipino immigrant women. The present study has several limitations. First, this study is a cross-sectional study, which may not yield temporal relationships. Second, measurement errors inherent in dietary assessment or from one-day 24-hour recall cannot be ignored. Although we adjusted for potential confounding factors, the presence of residual or unmeasured confounding factors may not be ruled out. The specific inherited condition, such as familial hypercholesterolemia, was not assessed in our study. However, low prevalence of familial hypercholesterolemia [58] may suggest that its potential effect on our results was likely to be trivial. Interpretation should be cautious in generalizing the conclusions to the general Filipino population because this study was conducted among Filipino immigrant adult women in Korea.

## Conclusions

We found that high dietary SFA intake was significantly associated with a high prevalence of dyslipidemia, high TC, and LDL-C. A suggestive inverse association was found between dietary MUFA intake and the prevalence of dyslipidemia, but no associations were found for total fat or PUFA intake. We also found that the association between dietary SFA intake and dyslipidemia was more pronounced among individuals with homozygous C alleles of rs6102059 polymorphisms near *MAFB*. Further prospective cohort studies are warranted to replicate our findings in the Asian population. In our study, 28% of Filipino women did not meet the recommended saturated fat intake (≤7% of total energy intake [59,60]). Given the overall increase in SFA intake over time in Korea [10] and the similar SFA intake between Filipino women and Korean women [10], it is important to prioritize public health interventions to address the health needs of immigrants and lower their burden of chronic disease, which lead to improving the health of the overall population.

## Data accessibility statement

Datasets are not publicly available for confidentiality reasons, but available from corresponding author on reasonable request. The study overview is presented on the FiLWHEL study website (www.filwhel.org), and the data access committee (nutepid@gmail.com) is open for requesting the relevant data.

## Additional File

The additional file for this article can be found as follows:

10.5334/gh.1209.s1 Supplementary Tables. Supplementary Tables 1 to 6.
