# Supplementary material for: Associations of Dietary Intakes of Total and Specific Types of Fat with Blood Lipid Levels in the Filipino Women’s Diet and Health Study (FiLWHEL)
Source: Glob Heart. 2023 Jun 12;18(1):29. doi: 10.5334/gh.1209 (PMC10275172; doi:10.5334/gh.1209)
Supplement: Supplementary Tables. — Supplementary Tables 1 to 6. [file gh-18-1-1209-s1.pdf]

Chromosome	SNP	Alleles (minor<major)	MAF[a]	MAF[b]	The nearest or within genes
5q13	rs3846663	T<C	0.51	0.49	*HMGCR*
5q23	rs1501908	G<C	0.28	0.36	*TIMD4-HAVCR1*[c]
12q24	rs2650000	A<C	0.44	0.43	*HNF1A*
20q12	rs6102059	T<C	0.56	0.44	*MAFB*

Supplementary Table 1. SNPs selected in our study

Abbreviations: SNP, single nucleotide polymorphism; MAF, minor allele frequency.
[a]The MAFs of the variants in East Asian were obtained from the 1000 Genomes Project Phase 3 populations using Ensembl GRCh37 browser [1-3].
[b]The MAFs of the variants in the Filipino immigrant women in our study were presented.
[c]rs1501908 is interval spanned by *TIMD4* and *HAVCR1*.

References:

1. **Cunningham F, Allen JE, Allen J, Alvarez-Jarreta J, Amode M R, Armean Irina M, et al.** Ensembl 2022. Nucleic Acids Research. 2022; 50(D1): D988-D995. DOI: 10.1093/nar/gkab1049
2. **EMBL-EBI**. Ensembl GRCh37 Release 108 (Oct 2022). http://grch37.ensembl.org/index.html (accessed 21 January 2023).
3. **Auton A, Abecasis GR, Altshuler DM, Durbin RM, Abecasis GR, Bentley DR, et al.** A global reference for human genetic variation. Nature. 2015; 526(7571): 68-74. DOI: 10.1038/nature15393

	Total fat			Saturated fat			Monounsaturated fat			Polyunsaturated fat		
	Tertile 1	Tertile 2	Tertile 3	Tertile 1	Tertile 2	Tertile 3	Tertile 1	Tertile 2	Tertile 3	Tertile 1	Tertile 2	Tertile 3
**N**	135	136	135	135	136	135	135	136	135	135	136	135
**Mean (SD)[a]**												
Age (years)	36.57 (7.94)	35.31 (7.20)	33.64 (8.25)	36.23 (7.99)	35.46 (7.83)	33.81 (7.68)	35.94 (8.39)	35.69 (7.52)	33.88 (7.59)	35.08 (7.71)	35.38 (7.72)	35.05 (8.25)
BMI (kg/m^2)	23.83 (4.04)	23.83 (4.03)	23.21 (3.37)	24.03 (3.80)	23.44 (3.73)	23.40 (3.95)	23.75 (3.82)	23.58 (3.72)	23.54 (3.97)	23.66 (3.92)	23.8 (4.06)	23.53 (3.51)
Total energy intake (kcal/day)	1689.60 (593.90)	1738.97 (660.09)	1836.37 (702.94)	1680.42 (610.68)	1865.74 (699.51)	1717.83 (641.65)	1689.64 (634.08)	1838.29 (637.32)	1736.28 (638.26)	1723.32 (677.15)	1805.02 (666.14)	1736.11 (622.54)
Carbohydrate (% of total energy)	67.67 (98.85)	57.72 (5.65)	45.30 (7.87)	61.32 (12.77)	58.46 (9.15)	50.90 (10.95)	60.87 (13.38)	59.57 (8.98)	50.23 (9.86)	59.23 (13.59)	58.51 (10.41)	52.95 (10.39)
Protein (% of total energy)	15.05 (5.04)	16.23 (4.18)	16.94 (4.26)	16.35 (5.17)	15.92 (4.24)	15.96 (4.25)	16.51 (5.76)	15.57 (3.83)	16.15 (3.82)	16.30 (5.27)	15.98 (4.50)	15.94 (3.84)
Total fat (% of total energy)	16.87 (4.46)	25.89 (2.28)	37.62 (5.87)	21.86 (9.03)	25.71 (7.45)	32.80 (8.85)	11.90 (9.23)	11.97 (6.57)	20.03 (8.86)	23.66 (10.15)	23.52 (7.57)	31.119 (9.28)
SFA (% of total energy)	3.20 (2.26)	5.08 (3.11)	8.29 (5.42)	1.57 (0.87)	4.56 (0.99)	10.46 (3.82)	2.34 (2.50)	4.51 (1.84)	9.72 (4.35)	3.15 (2.82)	5.49 (3.59)	7.93 (5.01)
MUFA (% of total energy)	3.31 (2.48)	5.42 (3.41)	9.23 (6.19)	2.24 (1.83)	5.22 (2.76)	10.50 (5.29)	1.47 (1.07)	4.88 (1.04)	11.62 (4.28)	2.43 (2.43)	5.86 (3.62)	9.66 (5.37)
PUFA (% of total energy)	2.57 (1.94)	3.85 (2.76)	5.58 (4.47)	2.37 (2.11)	3.95 (3.05)	5.68 (4.08)	1.61 (1.49)	4.00 (2.52)	6.38 (3.99)	0.98 (0.62)	3.22 (0.71)	7.80 (3.28)
**N (%)[b]**												
Length of Stay in Korea												
<5 years	24 (17.91)	32 (24.81)	49 (37.69)	33 (25.19)	30 (22.73)	42 (32.31)	34 (26.15)	26 (19.55)	45 (34.62)	34 (25.95)	28 (20.74)	43 (33.86)
≥5 years	110 (82.09)	97 (75.19)	81 (62.31)	98 (74.81)	102 (77.27)	88 (67.69)	96 (73.85)	107 (80.45)	85 (65.38)	97 (74.05)	107 (79.26)	84 (66.14)
Insulin resistance												
No	108 (80.00)	97 (71.32)	100 (74.07)	107 (79.26)	103 (75.74)	95 (70.37)	106 (78.52)	107 (78.68)	92 (68.15)	105 (77.78)	104 (76.47)	96 (71.11)
Yes	27 (20.00)	39 (28.68)	35 (24.93)	28 (20.74)	33 (24.26)	40 (29.63)	29 (21.48)	29 (21.32)	43 (31.85)	30 (22.22)	32 (23.53)	39 (28.89)
Employment status												
Employed	61 (45.19)	61 (44.85)	57 (42.86)	56 (41.48)	67 (49.63)	56 (41.79)	56 (41.79)	63 (46.32)	60 (44.78)	53 (39.85)	67 (49.26)	59 (43.70)
Unemployed	74 (54.81)	75 (55.15)	76 (57.14)	79 (58.52)	68 (50.37)	78 (58.21)	78 (58.21)	73 (53.68)	74 (55.22)	80 (60.15)	69 (50.74)	76 (56.30)

Supplementary Table 2. Baseline characteristics according to % energy from total fat and specific types of fats

Abbreviations: BMI, body mass index; SFA, saturated fat; MUFA, monounsaturated fat; PUFA, polyunsaturated fat.
There were missing data for a few participants; BMI (n=2), length of stay in Korea (n=13), and occupation (n=2).
[a]Mean (SD) for continuous variables.
[b]n (%) for categorical variables.

	Tertiles of energy-adjusted dietary fat intake				
	**Tertile 1**	**Tertile 2**	**Tertile 3**	***P for trend***	**Per 10g of fat intake[d]**
**Total Fat**					
**Dyslipidemia**					
Median intake[a], g/day	35.09	50.27	72.7		
No. of cases/total	43/135	47/136	40/135		
Age- and energy-adjusted model[b]	1.00	1.20 (0.72-2.03)	1.09 (0.64-1.87)	0.79	1.06 (0.94-1.19)
Multivariate model[c]	1.00	1.21 (0.70-2.09)	1.19 (0.68-2.11)	0.57	1.10 (0.97-1.24)
**TC ≥200 mg/dL**					
No. of cases/total	33/127	36/127	31/127		
Age- and energy-adjusted model[b]	1.00	1.18 (0.67-2.07)	1.15 (0.63-2.07)	0.68	1.10 (0.96-1.25)
Multivariate model[c]	1.00	1.14 (0.63-2.06)	1.28 (0.69-2.39)	0.44	1.14 (1.00-1.31)
**TG ≥150 mg/dL**					
No. of cases/total	12/127	13/127	11/127		
Age- and energy-adjusted model[b]	1.00	1.14 (0.49-2.61)	1.08 (0.45-2.60)	0.88	1.00 (0.83-1.21)
Multivariate model[c]	1.00	1.18 (0.50-2.79)	1.44 (0.57-3.61)	0.44	1.08 (0.88-1.32)
**LDL-C ≥130 mg/dL**					
No. of cases/total	20/127	25/127	24/127		
Age- and energy-adjusted model[b]	1.00	1.38 (0.71-2.66)	1.57 (0.80-3.09)	0.21	1.11 (0.96-1.29)
Multivariate model[c]	1.00	1.44 (0.72-2.89)	1.84 (0.90-3.79)	0.10	1.19 (1.02-1.40)
**HDL-C <50 mg/dL**					
No. of cases/total	38/127	36/127	32/127		
Age- and energy-adjusted model[b]	1.00	0.92 (0.53-1.58)	0.72 (0.41-1.26)	0.24	0.88 (0.78-1.00)
Multivariate model[c]	1.00	1.01 (0.56-1.79)	0.79 (0.43-1.46)	0.43	0.90 (0.79-1.03)
**SFA**					
**Dyslipidemia**					
Median intake[a], g/day	3.37	8.96	18.39		
No. of cases/total	38/135	49/136	43/135		
Age- and energy-adjusted model[b]	1.00	1.48 (0.87-2.51)	1.43 (0.83-2.47)	0.24	1.23 (0.96-1.58)
Multivariate model[c]	1.00	1.62 (0.92-2.84)	1.54 (0.87-2.72)	0.19	1.28 (0.99-1.67)
**TC ≥200 mg/dL**					
No. of cases/total	25/127	38/127	37/127		
Age- and energy-adjusted model[b]	1.00	1.78 (0.98-3.23)	2.13 (1.16-3.92)	0.02	1.41 (1.08-1.86)
Multivariate model[c]	1.00	1.99 (1.06-3.74)	2.35 (1.25-4.43)	0.01	1.48 (1.11-1.97)
**TG ≥150 mg/dL**					
No. of cases/total	11/127	16/127	9/127		
Age- and energy-adjusted model	1.00	1.55 (0.68-3.52)	0.93 (0.37-2.37)	0.78	1.00 (0.65-1.53)
Multivariate model	1.00	1.67 (0.71-3.94)	0.99 (0.38-2.59)	0.89	1.06 (0.68-1.66)
**LDL-C ≥130 mg/dL**					
No. of cases/total	17/127	26/127	26/127		
Age- and energy-adjusted model[b]	1.00	1.67 (0.84-3.31)	2.08 (1.04-4.15)	0.05	1.44 (1.07-1.95)
Multivariate model[c]	1.00	2.10 (1.01-4.35)	2.52 (1.21-5.26)	0.02	1.55 (1.13-2.14)
**HDL-C <50 mg/dL**					

No. of cases/total	39/127	34/127	33/127		
Age- and energy-adjusted model[b]	1.00	0.82 (0.48-1.43)	0.73 (0.42-1.28)	0.29	0.95 (0.73-1.24)
Multivariate model[c]	1.00	0.97 (0.54-1.73)	0.77 (0.43-1.39)	0.37	0.98 (0.74-1.30)
**MUFA**					
**Dyslipidemia**					
Median intake[a], g/day	2.47	9.41	22.92		
No. of cases/total	46/135	46/136	38/135		
Age- and energy-adjusted model[b]	1.00	0.97 (0.58-1.62)	0.90 (0.52-1.56)	0.69	1.04 (0.87-1.24)
Multivariate model[c]	1.00	0.99 (0.57-1.72)	0.85 (0.48-1.52)	0.57	1.04 (0.86-1.25)
**TC ≥200 mg/dL**					
No. of cases/total	33/127	33/127	34/127		
Age- and energy-adjusted model[b]	1.00	0.98 (0.55-1.73)	1.33 (0.73-2.43)	0.32	1.19 (0.98-1.44)
Multivariate model[c]	1.00	0.98 (0.54-1.78)	1.28 (0.69-2.40)	0.40	1.21 (0.99-1.48)
**TG ≥150 mg/dL**					
No. of cases/total	14/127	11/127	11/127		
Age- and energy-adjusted model[b]	1.00	0.74 (0.32-1.70)	0.85 (0.35-2.05)	0.77	1.09 (0.83-1.42)
Multivariate model[c]	1.00	0.69 (0.29-1.66)	0.77 (0.31-1.92)	0.64	1.11 (0.83-1.48)
**LDL-C ≥130 mg/dL**					
No. of cases/total	24/127	21/127	24/127		
Age- and energy-adjusted model[b]	1.00	0.83 (0.43-1.60)	1.27 (0.64-2.49)	0.41	1.21 (0.98-1.50)
Multivariate model[c]	1.00	0.80 (0.40-1.61)	1.31 (0.65-2.65)	0.37	1.27 (1.02-1.59)
**HDL-C <50 mg/dL**					
No. of cases/total	38/127	31/127	37/127		
Age- and energy-adjusted model[b]	1.00	0.76 (0.44-1.33)	0.89 (0.51-1.58)	0.80	1.01 (0.84-1.22)
Multivariate model[c]	1.00	0.76 (0.42-1.38)	0.89 (0.49-1.63)	0.81	1.01 (0.83-1.22)
**PUFA**					
**Dyslipidemia**					
Median intake[a], g/day	1.94	6.16	14.16		
No. of cases/total	46/135	41/136	43/135		
Age- and energy-adjusted model[b]	1.00	0.80 (0.47-1.35)	1.00 (0.59-1.70)	0.89	1.07 (0.81-1.42)
Multivariate model[c]	1.00	0.78 (0.44-1.36)	1.04 (0.59-1.83)	0.84	1.13 (0.84-1.54)
**TC ≥200 mg/dL**					
No. of cases/total	34/127	31/127	35/127		
Age- and energy-adjusted model[b]	1.00	0.88 (0.50-1.57)	1.17 (0.66-2.09)	0.51	1.22 (0.91-1.62)
Multivariate model[c]	1.00	0.83 (0.45-1.53)	1.18 (0.64-2.18)	0.49	1.31 (0.96-1.78)
**TG ≥150 mg/dL**					
No. of cases/total	11/127	10/127	15/127		
Age- and energy-adjusted model[b]	1.00	0.90 (0.37-2.21)	1.51 (0.65-3.52)	0.27	1.06 (0.69-1.62)
Multivariate model[c]	1.00	0.89 (0.35-2.28)	1.53 (0.63-3.69)	0.27	1.10 (0.69-1.75)
**LDL-C ≥130 mg/dL**					
No. of cases/total	25/127	21/127	23/127		
Age- and energy-adjusted model[b]	1.00	0.81 (0.42-1.55)	1.00 (0.52-1.92)	0.92	1.16 (0.84-1.61)
Multivariate model[c]	1.00	0.89 (0.45-1.77)	1.12 (0.56-2.24)	0.69	1.31 (0.92-1.85)

**HDL-C <50 mg/dL**					
No. of cases/total	37/127	32/127	37/127		
Age- and energy-adjusted model[b]	1.00	0.82 (0.47-1.43)	0.96 (0.55-1.67)	0.98	0.89 (0.67-1.19)
Multivariate model[c]	1.00	0.80 (0.44-1.46)	0.96 (0.53-1.74)	0.99	0.91(0.67-1.23)

Supplementary Table 3. Odds ratio (OR)s and 95% confidence interval (CI)s for dyslipidemia and lipid biomarkers according to dietary fat intake (no substitution model)

Abbreviations: SFA, saturated fat; MUFA, monounsaturated fat; PUFA, polyunsaturated fat; TC, total cholesterol; TG, triglyceride; LDL-C, LDL cholesterol; HDL-C, HDL cholesterol.
Dyslipidemia was defined as total cholesterol ≥200 mg/dL or triglyceride ≥150 mg/dL or LDL cholesterol ≥130 mg/dL.
Estimates are presented as odds ratios (OR)s and 95% confidence interval (CI).
[a]Median (g/day) of energy-adjusted fat and fat subtype intakes were calculated.
[b]Model was adjusted for age (years, continuous) and total energy intake (kcal/day, continuous).
[c]Model was adjusted for age (years, continuous), total energy intake (kcal/day, continuous), BMI (<18.5, 18.5-<23, 23-<25, ≥25 kg/m^2), length of stay in Korea (<5 years, ≥5 years), employment status (employed, unemployed), region (Seoul, Incheon/Gyeonggido, Daejeon/Chuncheongnamdo), vigorous activity (no, yes) and educational level (elementary or high school, association or vocational or college or above).
[d]For 10 g/day increment of each type of fat.

	Tertiles of % energy from dietary fat intake				
	Tertile 1	Tertile 2	Tertile 3	*P for trend*	Per 5% energy from fat intake[d]
**Total Fat**					
**Dyslipidemia**					
Median intake[a], % energy/day	18.23	25.56	36.01		
No. of cases/total	42/135	48/136	40/135		
Age- and energy-adjusted model[b]	1.00	1.30 (0.77-2.21)	1.10 (0.63-1.91)	0.81	1.07 (0.95-1.20)
Multivariate model[c]	1.00	1.26 (0.72-2.20)	1.12 (0.62-2.00)	0.76	1.10 (0.97-1.25)
**TC ≥200 mg/dL**					
No. of cases/total	33/127	36/127	31/127		
Age- and energy-adjusted model[b]	1.00	1.15 (0.65-2.06)	1.05 (0.58-1.93)	0.90	1.09 (0.96-1.24)
Multivariate model[c]	1.00	1.09 (0.59-1.99)	1.12 (0.60-2.12)	0.72	1.14 (0.99-1.30)
**TG ≥150 mg/dL**					
No. of cases/total	14/127	12/127	10/127		
Age- and energy-adjusted model[b]	1.00	0.90 (0.39-2.09)	0.85 (0.35-2.08)	0.72	1.02 (0.84-1.24)
Multivariate model[c]	1.00	0.88 (0.37-2.11)	1.01 (0.40-2.55)	0.97	1.09 (0.88-1.33)
**LDL-C ≥130 mg/dL**					
No. of cases/total	21/127	24/127	24/127		
Age- and energy-adjusted model[b]	1.00	1.22 (0.62-2.39)	1.38 (0.70-2.75)	0.36	1.12 (0.96-1.29)
Multivariate model[c]	1.00	1.22 (0.60-2.48)	1.51 (0.73-3.12)	0.27	1.19 (1.01-1.39)
**HDL-C <50 mg/dL**					
No. of cases/total	39/127	38/127	29/127		
Age- and energy-adjusted model[b]	1.00	0.97 (0.56-1.68)	0.65 (0.36-1.16)	0.13	0.90 (0.79-1.02)
Multivariate model[c]	1.00	1.00 (0.56-1.78)	0.65 (0.35-1.21)	0.16	0.92 (0.81-1.06)
**SFA**					
**Dyslipidemia**					
Median intake[a], % energy/day	1.66	4.6	9.34		
No. of cases/total	39/135	46/136	45/135		
Age- and energy-adjusted model[b]	1.00	1.93 (1.01-3.68)	2.94 (1.33-6.50)	0.01	1.80 (1.13-2.88)
Multivariate model[c]	1.00	2.20 (1.11-4.37)	3.69 (1.57-8.68)	0.004	2.12 (1.28-3.53)
**TC ≥200 mg/dL**					
No. of cases/total	27/127	37/127	36/127		
Age- and energy-adjusted model[b]	1.00	2.58 (1.24-5.40)	3.90 (1.60-9.48)	0.005	1.78 (1.07-2.94)
Multivariate model[c]	1.00	3.12 (1.43-6.82)	5.23 (2.00-13.70)	0.002	2.00 (1.17-3.43)
**TG ≥150 mg/dL**					
No. of cases/total	13/127	17/127	6/127		
Age- and energy-adjusted model	1.00	1.04 (0.37-2.89)	0.17 (0.04-0.71)	0.01	0.60 (0.27-1.33)
Multivariate model	1.00	1.09 (0.37-3.21)	0.18 (0.04-0.83)	0.01	0.65 (0.28-1.55)
**LDL-C ≥130 mg/dL**					
No. of cases/total	17/127	26/127	26/127		

Age- and energy-adjusted model[b]	1.00	2.95 (1.26-6.90)	4.05 (1.48-11.04)	0.01	1.89 (1.08-3.29)
Multivariate model[c]	1.00	3.85 (1.54-9.63)	5.60 (1.88-16.72)	0.004	2.20 (1.19-4.05)
**HDL-C <50 mg/dL**					
No. of cases/total	40/127	37/127	29/127		
Age- and energy-adjusted model[b]	1.00	0.75 (0.38-1.47)	0.30 (0.13-0.74)	0.01	0.80 (0.49-1.29)
Multivariate model[c]	1.00	0.81 (0.39-1.66)	0.31 (0.12-0.80)	0.01	0.84 (0.50-1.42)
**MUFA**					
**Dyslipidemia**					
Median intake[a], % energy/day	1.55	4.95	10.7		
No. of cases/total	47/135	44/136	39/135		
Age- and energy-adjusted model[b]	1.00	0.55 (0.28-1.09)	0.33 (0.13-0.81)	0.02	0.64 (0.40-1.02)
Multivariate model[c]	1.00	0.55 (0.26-1.14)	0.28 (0.11-0.72)	0.01	0.55 (0.33-0.91)
**TC ≥200 mg/dL**					
No. of cases/total	35/127	34/127	31/127		
Age- and energy-adjusted model[b]	1.00	0.52 (0.24-1.12)	0.32 (0.12-0.86)	0.03	0.74 (0.45-1.23)
Multivariate model[c]	1.00	0.51 (0.22-1.17)	0.26 (0.09-0.75)	0.01	0.67 (0.39-1.16)
**TG ≥150 mg/dL**					
No. of cases/total	12/127	11/127	13/127		
Age- and energy-adjusted model[b]	1.00	0.93 (0.29-3.01)	3.38 (0.78-14.65)	0.06	1.76 (0.85-3.66)
Multivariate model[c]	1.00	0.95 (0.28-3.25)	3.24 (0.71-14.73)	0.08	1.70 (0.76-3.84)
**LDL-C ≥130 mg/dL**					
No. of cases/total	24/127	22/127	23/127		
Age- and energy-adjusted model[b]	1.00	0.50 (0.21-1.19)	0.44 (0.15-1.29)	0.17	0.73 (0.41-1.28)
Multivariate model[c]	1.00	0.49 (0.19-1.23)	0.37 (0.12-1.18)	0.12	0.64 (0.34-1.21)
**HDL-C <50 mg/dL**					
No. of cases/total	38/127	30/127	38/127		
Age- and energy-adjusted model[b]	1.00	0.92 (0.43-1.95)	2.12 (0.80-5.62)	0.09	1.37 (0.85-2.20)
Multivariate model[c]	1.00	0.98 (0.44-2.20)	2.19 (0.78-6.16)	0.09	1.32 (0.79-2.21)
**PUFA**					
**Dyslipidemia**					
Median intake[a], % energy/day	1.01	3.26	6.75		
No. of cases/total	44/135	41/136	45/135		
Age- and energy-adjusted model[b]	1.00	0.99 (0.55-1.78)	1.31 (0.67-2.57)	0.37	1.13 (0.77-1.68)
Multivariate model[c]	1.00	1.04 (0.55-1.96)	1.27 (0.62-2.6)	0.52	1.25 (0.82-1.92)
**TC ≥200 mg/dL**					
No. of cases/total	33/127	32/127	35/127		
Age- and energy-adjusted model[b]	1.00	1.06 (0.55-2.02)	1.26 (0.60-2.64)	0.52	1.14 (0.75-1.72)
Multivariate model[c]	1.00	1.09 (0.54-2.17)	1.21 (0.55-2.66)	0.64	1.23 (0.78-1.94)
**TG ≥150 mg/dL**					
No. of cases/total	11/127	11/127	14/127		
Age- and energy-adjusted model[b]	1.00	1.00 (0.36-2.73)	1.23 (0.40-3.76)	0.67	0.89 (0.47-1.68)
Multivariate model[c]	1.00	1.05 (0.37-3.01)	1.18 (0.37-3.72)	0.76	0.90 (0.44-1.83)

**LDL-C ≥130 mg/dL**					
No. of cases/total	23/127	24/127	22/127		
Age- and energy-adjusted model[b]	1.00	1.09 (0.53-2.25)	0.89 (0.38-2.07)	0.71	1.05 (0.65-1.68)
Multivariate model[c]	1.00	1.23 (0.56-2.72)	0.89 (0.36-2.18)	0.65	1.17 (0.69-1.98)
**HDL-C <50 mg/dL**					
No. of cases/total	39/127	27/127	40/127		
Age- and energy-adjusted model[b]	1.00	0.63 (0.33-1.20)	1.10 (0.54-2.22)	0.50	0.74 (0.48-1.13)
Multivariate model[c]	1.00	0.63 (0.32-1.24)	1.08 (0.51-2.28)	0.53	0.74 (0.47-1.18)

Supplementary Table 4. Odds ratio (OR)s and 95% confidence interval (CI)s for dyslipidemia and lipid biomarkers according to % energy from fat intake replacing carbohydrate intake

Abbreviations: SFA, saturated fat; MUFA, monounsaturated fat; PUFA, polyunsaturated fat; TC, total cholesterol; TG, triglyceride; LDL-C, LDL cholesterol; HDL-C, HDL cholesterol.
Dyslipidemia was defined as total cholesterol ≥200 mg/dL or triglyceride ≥150 mg/dL or LDL cholesterol ≥130 mg/dL.
Estimates are presented as odds ratios (OR)s and 95% confidence interval (CI).
[a]Median of % energy from fat and fat subtype intakes were calculated.
[b]Model was adjusted for age (years, continuous) and total energy intake (kcal/day, continuous), percent energy from protein (tertile), and percent energy from other types of fats (tertile).
[c]Model was adjusted for age (years, continuous), total energy intake (kcal/day, continuous), BMI (<18.5, 18.5-<23, 23-<25, ≥25 kg/m^2), length length of stay in Korea (<5 years, ≥5 years), employment status (employed, unemployed), region (Seoul, Incheon/Gyeonggido, Daejeon/Chuncheongnamdo), vigorous activity (no, yes), educational level (elementary or high school, association or vocational or college or above), percent energy from protein (tertile), and percent energy from other types of fats (tertile).
[d]For 5% energy from fat per day increment of each type of fat. Energy from protein and other types of fats were included in the model as continuous variables (g/day).

	Tertiles of energy-adjusted dietary fat intake				
	Tertile 1	Tertile 2	Tertile 3	*P for trend*	Per 10g of fat intake[d]
**Total Fat**					
**Dyslipidemia**					
Median intake[a], g/day	35.10	50.27	72.71		
No. of cases/total	39/122	38/120	36/122		
Age- and energy-adjusted model[b]	1.00	1.05 (0.60-1.86)	1.19 (0.66-2.13)	0.56	1.11 (0.98-1.27)
Multivariate model[c]	1.00	1.07 (0.59-1.94)	1.39 (0.75-2.60)	0.28	1.17 (1.02-1.34)
**TC ≥200 mg/dL**					
No. of cases/total	31/120	33/120	31/124		
Age- and energy-adjusted model[b]	1.00	1.11 (0.61-2.02)	1.22 (0.66-2.26)	0.54	1.13 (0.99-1.30)
Multivariate model[c]	1.00	1.11 (0.60-2.08)	1.36 (0.71-2.60)	0.35	1.18 (1.02-1.36)
**TG ≥150 mg/dL**					
No. of cases/total	12/120	11/120	10/124		
Age- and energy-adjusted model[b]	1.00	1.00 (0.42-2.42)	1.05 (0.42-2.64)	0.92	1.00 (0.82-1.23)
Multivariate model[c]	1.00	1.04 (0.42-2.59)	1.28 (0.49-3.31)	0.61	1.06 (0.85-1.31)
**LDL-C ≥130 mg/dL**					
No. of cases/total	20/120	23/120	24/124		
Age- and energy-adjusted model[b]	1.00	1.23 (0.62-2.44)	1.56 (0.78-3.14)	0.21	1.14 (0.98-1.33)
Multivariate model[c]	1.00	1.32 (0.64-2.71)	1.79 (0.86-3.74)	0.12	1.20 (1.02-1.42)
**HDL-C <50 mg/dL**					
No. of cases/total	36/120	34/120	31/124		
Age- and energy-adjusted model[b]	1.00	0.96 (0.54-1.68)	0.76 (0.42-1.37)	0.35	0.90 (0.79-1.03)
Multivariate model[c]	1.00	1.04 (0.57-1.89)	0.84 (0.45-1.57)	0.56	0.92 (0.80-1.06)
**SFA**					
**Dyslipidemia**					
Median intake[a], g/day	3.28	8.91	18.32		
No. of cases/total	32/121	44/122	37/121		
Age- and energy-adjusted model[b]	1.00	2.22 (1.14-4.32)	2.41 (1.05-5.52)	0.07	1.50 (1.00-2.27)
Multivariate model[c]	1.00	2.76 (1.36-5.60)	3.21 (1.33-7.79)	0.02	1.73 (1.11-2.69)
**TC ≥200 mg/dL**					
No. of cases/total	22/121	36/119	37/124		
Age- and energy-adjusted model[b]	1.00	2.63 (1.28-5.39)	3.61 (1.52-8.57)	0.01	1.58 (1.04-2.41)
Multivariate model[c]	1.00	3.33 (1.55-7.14)	4.65 (1.88-11.48)	0.003	1.71 (1.10-2.67)
**TG ≥150 mg/dL**					
No. of cases/total	11/121	14/119	8/124		
Age- and energy-adjusted model	1.00	1.57 (0.59-4.17)	0.87 (0.23-3.26)	0.70	0.79 (0.39-1.59)
Multivariate model	1.00	1.86 (0.66-5.26)	1.14 (0.30-4.30)	0.99	0.88 (0.42-1.81)
**LDL-C ≥130 mg/dL**					
No. of cases/total	17/121	24/119	26/124		
Age- and energy-adjusted model[b]	1.00	2.04 (0.93-4.51)	2.96 (1.14-7.70)	0.04	1.47 (0.93-2.34)
Multivariate model[c]	1.00	2.89 (1.23-6.76)	4.08 (1.48-11.21)	0.01	1.59 (0.96-2.62)
**HDL-C <50 mg/dL**					

No. of cases/total	38/121	31/119	32/124		
Age- and energy-adjusted model[b]	1.00	0.67 (0.35-1.30)	0.53 (0.24-1.19)	0.15	0.84 (0.56-1.25)
Multivariate model[c]	1.00	0.85 (0.42-1.70)	0.61 (0.26-1.43)	0.24	0.91 (0.59-1.40)
**MUFA**					
**Dyslipidemia**					
Median intake[a], g/day	2.37	9.65	23.37		
No. of cases/total	41/121	36/118	36/125		
Age- and energy-adjusted model[b]	1.00	0.60 (0.29-1.25)	0.55 (0.20-1.50)	0.36	0.90 (0.64-1.27)
Multivariate model[c]	1.00	0.57 (0.26-1.24)	0.40 (0.14-1.15)	0.12	0.82 (0.56-1.19)
**TC ≥200 mg/dL**					
No. of cases/total	31/121	30/118	34/125		
Age- and energy-adjusted model[b]	1.00	0.67 (0.31-1.47)	0.68 (0.23-2.00)	0.66	0.93 (0.66-1.30)
Multivariate model[c]	1.00	0.65 (0.29-1.46)	0.56 (0.18-1.67)	0.39	0.87 (0.61-1.25)
**TG ≥150 mg/dL**					
No. of cases/total	14/121	8/118	11/125		
Age- and energy-adjusted model[b]	1.00	0.33 (0.10-1.08)	0.43 (0.09-2.07)	0.56	1.26 (0.79-2.01)
Multivariate model[c]	1.00	0.30 (0.09-1.00)	0.32 (0.07-1.50)	0.30	1.22 (0.73-2.05)
**LDL-C ≥130 mg/dL**					
No. of cases/total	24/121	19/118	24/125		
Age- and energy-adjusted model[b]	1.00	0.61 (0.25-1.45)	0.75 (0.23-2.45)	0.89	0.99 (0.69-1.43)
Multivariate model[c]	1.00	0.49 (0.19-1.22)	0.55 (0.16-1.87)	0.57	0.95 (0.64-1.42)
**HDL-C <50 mg/dL**					
No. of cases/total	36/121	28/118	37/125		
Age- and energy-adjusted model[b]	1.00	0.88 (0.43-1.83)	1.32 (0.48-3.65)	0.44	1.20 (0.88-1.64)
Multivariate model[c]	1.00	0.80 (0.37-1.72)	1.16 (0.40-3.37)	0.61	1.12 (0.81-1.55)
**PUFA**					
**Dyslipidemia**					
Median intake[a], g/day	2.05	6.26	14.41		
No. of cases/total	44/128	31/117	38/119		
Age- and energy-adjusted model[b]	1.00	0.71 (0.36-1.38)	1.11 (0.52-2.39)	0.50	1.05 (0.73-1.50)
Multivariate model[c]	1.00	0.67 (0.32-1.40)	1.28 (0.56-2.94)	0.32	1.130(0.76-1.67)
**TC ≥200 mg/dL**					
No. of cases/total	33/123	29/121	33/120		
Age- and energy-adjusted model[b]	1.00	0.77 (0.37-1.60)	0.95 (0.41-2.23)	0.87	1.14 (0.79-1.63)
Multivariate model[c]	1.00	0.69 (0.31-1.50)	0.98 (0.40-2.43)	0.73	1.23 (0.83-1.82)
**TG ≥150 mg/dL**					
No. of cases/total	11/123	9/121	13/120		
Age- and energy-adjusted model[b]	1.00	1.34 (0.45-3.96)	2.77 (0.75-10.14)	0.10	0.91 (0.52-1.59)
Multivariate model[c]	1.00	1.33 (0.43-4.14)	3.00 (0.79-11.41)	0.08	0.90 (0.48-1.69)
**LDL-C ≥130 mg/dL**					
No. of cases/total	25/123	20/121	22/120		
Age- and energy-adjusted model[b]	1.00	0.73 (0.32-1.64)	0.80 (0.31-2.09)	0.82	1.02 (0.68-1.53)
Multivariate model[c]	1.00	0.81 (0.34-1.94)	0.93 (0.34-2.59)	0.98	1.12 (0.72-1.76)

**HDL-C <50 mg/dL**					
No. of cases/total	35/123	30/121	36/120		
Age- and energy-adjusted model[b]	1.00	0.88 (0.45-1.72)	1.15 (0.52-2.57)	0.58	0.83 (0.58-1.20)
Multivariate model[c]	1.00	0.87 (0.43-1.77)	1.18 (0.51-2.74)	0.56	0.86 (0.59-1.27)

Supplementary Table 5. Odds ratio (OR)s and 95% confidence interval (CI)s for dyslipidemia and lipid biomarkers according to dietary fat intake replacing carbohydrate intake among participants without history of metabolic disorders

Abbreviations: SFA, saturated fat; MUFA, monounsaturated fat; PUFA, polyunsaturated fat; TC, total cholesterol; TG, triglyceride; LDL-C, LDL cholesterol; HDL-C, HDL cholesterol.
Dyslipidemia was defined as total cholesterol ≥200 mg/dL or triglyceride ≥150 mg/dL or LDL cholesterol ≥130 mg/dL.
Estimates are presented as odds ratios (OR)s and 95% confidence interval (CI).
[a]Median (g/day) of energy-adjusted fat and fat subtype intakes were calculated.
[b]Model was adjusted for age (years, continuous), total energy intake (kcal/day, continuous), energy-adjusted protein (tertile), and energy-adjusted other types of fats (tertile).
[c]Model was adjusted for age (years, continuous), total energy intake (kcal/day, continuous), BMI (<18.5, 18.5-<23, 23-<25, ≥25 kg/m^2), length length of stay in Korea (<5 years, ≥5 years), employment status (employed, unemployed), region (Seoul, Incheon/Gyeonggido, Daejeon/Chuncheongnamdo), vigorous activity (no, yes), educational level (elementary or high school, association or vocational or college or above), energy-adjusted protein (tertile), and energy-adjusted other types of fats (tertile).
[d]For 10 g/day increment of each type of fat. Energy-adjusted protein and other types of fats were included in the model as continuous variables (g/day).

	Tertiles of energy-adjusted dietary fat intake				
	**Tertile 1**	**Tertile 2**	**Tertile 3**	***P for trend***	**Per 10g of fat intake[a]**
**Total Fat**					
**Dyslipidemia**	1.00	1.15 (0.67-1.97)	1.10 (0.62-1.94)	0.78	1.07 (0.94-1.21)
**TC ≥200 mg/dL**	1.00	1.08 (0.60-1.95)	1.14 (0.61-2.13)	0.69	1.11 (0.96-1.27)
**TG ≥150 mg/dL**	1.00	1.11 (0.46-2.70)	1.25 (0.48-3.24)	0.64	1.04 (0.84-1.28)
**LDL-C ≥130 mg/dL**	1.00	1.38 (0.69-2.75)	1.69 (0.82-3.46)	0.16	1.17 (0.998-1.36)
**HDL-C <50 mg/dL**	1.00	0.95 (0.54-1.67)	0.70 (0.38-1.27)	0.23	0.90 (0.79-1.02)
**SFA**					
**Dyslipidemia**	1.00	1.98 (1.06-3.70)	2.45 (1.13-5.30)	0.04	1.60 (1.07-2.39)
**TC ≥200 mg/dL**	1.00	2.42 (1.20-4.87)	3.25 (1.40-7.53)	0.01	1.52 (0.998-2.30)
**TG ≥150 mg/dL**	1.00	1.88 (0.69-5.14)	1.31 (0.37-4.62)	0.83	0.87 (0.43-1.75)
**LDL-C ≥130 mg/dL**	1.00	2.67 (1.19-5.98)	3.55 (1.36-9.29)	0.02	1.51 (0.94-2.42)
**HDL-C <50 mg/dL**	1.00	0.82 (0.42-1.58)	0.62 (0.27-1.40)	0.88	0.91 (0.60-1.38)
**MUFA**					
**Dyslipidemia**	1.00	0.62 (0.31-1.23)	0.4 (0.16-1.02)	0.07	0.77 (0.55-1.08)
**TC ≥200 mg/dL**	1.00	0.61 (0.28-1.31)	0.53 (0.19-1.50)	0.32	0.91 (0.64-1.28)
**TG ≥150 mg/dL**	1.00	0.29 (0.09-0.93)	0.22 (0.05-1.01)	0.14	1.21 (0.72-2.02)
**LDL-C ≥130 mg/dL**	1.00	0.51 (0.21-1.22)	0.58 (0.18-1.84)	0.61	1.00 (0.68-1.46)
**HDL-C <50 mg/dL**	1.00	0.76 (0.37-1.58)	1.16 (0.42-3.20)	0.55	1.14 (0.83-1.57)
**PUFA**					
**Dyslipidemia**	1.00	0.90 (0.48-1.68)	1.11 (0.53-2.32)	0.69	1.07 (0.74-1.53)
**TC ≥200 mg/dL**	1.00	0.89 (0.44-1.82)	1.02 (0.45-2.35)	0.85	1.12 (0.77-1.63)
**TG ≥150 mg/dL**	1.00	1.74 (0.58-5.23)	3.09 (0.86-11.20)	0.09	0.87 (0.48-1.60)
**LDL-C ≥130 mg/dL**	1.00	0.92 (0.41-2.07)	0.87 (0.34-2.25)	0.80	1.00 (0.65-1.55)
**HDL-C <50 mg/dL**	1.00	0.98 (0.50-1.90)	1.01 (0.46-2.23)	0.96	0.77 (0.53-1.13)

Supplementary Table 6. Odds ratio (OR)s and 95% confidence interval (CI)s for dyslipidemia and lipid biomarkers according to dietary fat intake replacing carbohydrate intake (adjusted for the waist to hip ratio)

Abbreviations: SFA, saturated fat; MUFA, monounsaturated fat; PUFA, polyunsaturated fat; TC, total cholesterol; TG, triglyceride; LDL-C, LDL cholesterol; HDL-C, HDL cholesterol.
Dyslipidemia was defined as total cholesterol ≥200 mg/dL or triglyceride ≥150 mg/dL or LDL cholesterol ≥130 mg/dL.
Estimates are presented as odds ratios (OR)s and 95% confidence interval (CI).
Model was adjusted for age (years, continuous), total energy intake (kcal/day, continuous), waist to hip ratio (<0.85, ≥0.85), length length of stay in Korea (<5 years, ≥5 years), employment status (employed, unemployed), region (Seoul, Incheon/Gyeonggido, Daejeon/Chuncheongnamdo), vigorous activity (no, yes), educational level (elementary or high school, association or vocational or college or above), energy-adjusted protein (tertile), and energy-adjusted other types of fats (tertile).
[a]For 10 g/day increment of each type of fat. Energy-adjusted protein and other types of fats were included in the model as continuous variables (g/day).
